# Current Advances in the Physiological Roles and Disease-Associated Mechanisms of ADAMTS16

**DOI:** 10.3390/ijms27157050

**Published:** 2026-08-06

**Authors:** Linxiu Wang, Jiazhen Zhang, Yuying Zhang

**Affiliations:** School of Biological Science and Technology, University of Jinan, Jinan 250022, China; 15536486276@163.com (L.W.); 17862253643@163.com (J.Z.)

**Keywords:** ADAMTS16, extracellular matrix, reproductive development, cancer, TGF-β signaling

## Abstract

As an important member of the ADAMTS (a disintegrin and metalloproteinase with thrombospondin motifs) family, ADAMTS16 participates in various physiological and pathological processes through the regulation of extracellular matrix (ECM) components. This review systematically delineates the expression profiles of ADAMTS16 in different tissues and cell types, as well as its diverse roles in reproductive development, cardiovascular diseases, ocular diseases, skeletal disorders, malignancies, and other pathological conditions, revealing significant differences in its expression patterns and mechanisms of action across various tissues. Cumulatively, current evidence suggests that ADAMTS16 may engage in direct or indirect interactions with ECM constituents to regulate cell proliferation, differentiation, and migration, and may also influence a range of cellular activities via synergistic cooperation with other cytokines.

## 1. Introduction

**Figure 1:**
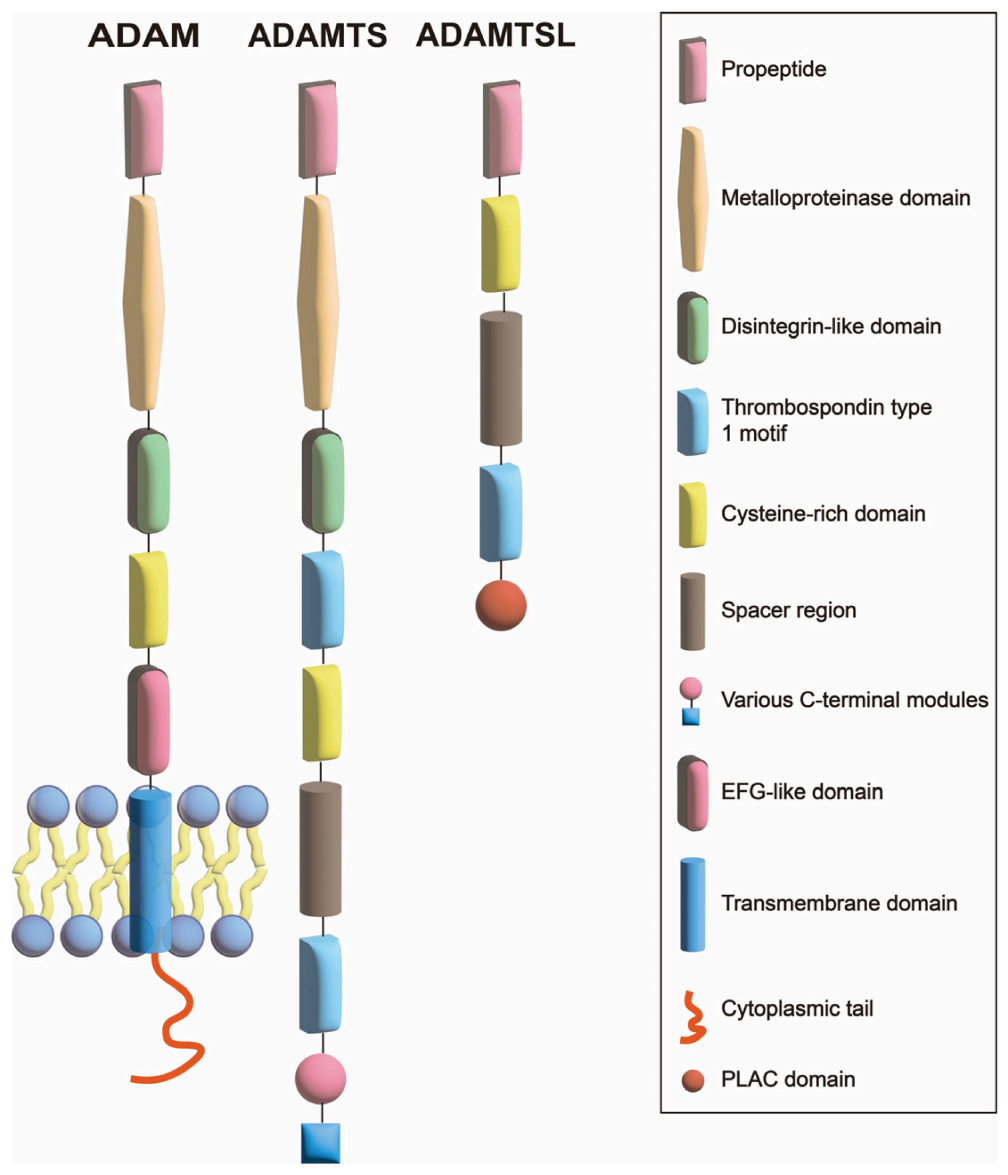
Domain structure schematics of ADAM, ADAMTS, and ADAMTSL. Since the signal peptide does not form part of the processed proteins, it is not depicted in the figure.

The first member of the ADAMTS (A Disintegrin and Metalloproteinase with Thrombospondin Motifs) family was first identified in 1997 [1]. Along with ADAM and ADAMTSL, it belongs to the zinc metalloproteinase superfamily. All three family members contain a signal peptide, a propeptide, a metalloproteinase domain, a disintegrin-like domain, and a cysteine-rich domain (Figure 1) [2,3]. Structurally, ADAM possesses a unique EGF-like domain, a transmembrane domain, and a cytoplasmic tail, and is primarily localized to the cell membrane. Approximately half of its members (e.g., ADAM8, 10, 12, 15, 17) possess catalytic activity, mediate the shedding of membrane-bound proteins, and are involved in cell adhesion, migration, and signaling regulation [4]. ADAMTS contains central thrombospondin type 1 repeats (TSRs). As secreted proteases, they are primarily responsible for the degradation and remodeling of the extracellular matrix, playing important roles in the maintenance of tissue homeostasis, inflammatory responses, and disease progression. ADAMTSL lacks the metalloproteinase domain but retains multiple thrombospondin type 1 repeats as well as a cysteine-rich/spacer domain, functioning in a non-enzymatic manner to participate in matrix assembly, cell adhesion, and microenvironmental stability. These three families are highly homologous in structure, and functionally distinct yet synergistic with one another, collectively constituting an important molecular network that regulates the extracellular microenvironment. Studies have reported that ADAM10 and ADAM17 are primarily responsible for the cleavage of various bioactive substrates, including cytokine receptors, adhesion molecules and growth factors [5,6,7,8]. Notably, the absence of ADAM15 leads to increased expression of integrin α7 protein while integrin β1 expression remains unchanged. This expression imbalance disrupts the interaction between cardiomyocyte integrin α7/β1 and laminin, thereby inducing cardiac hypertrophy in mice [9]. Functional studies of the ADAMTS family have primarily focused on their involvement in extracellular matrix remodeling, organ development, inflammatory regulation, cardiovascular diseases, cancer, and fibrotic diseases, among others [10,11,12,13,14]. Specifically, deficiency of ADAMTS6 leads to pulmonary hypoplasia, skeletal deformities, and congenital heart defects in the developing embryo [15]; ADAMTS4 expression is elevated in human atherosclerotic plaques and in the plasma of patients with acute coronary syndrome, where it compromises vascular structural integrity by degrading matrix components such as vascular wall proteoglycans [16]. ADAMTS8 is frequently silenced via promoter methylation in multiple tumor types, and its overexpression inhibits tumor cell proliferation, migration, and invasion by antagonizing the EGFR-MEK-ERK and Wnt/β-catenin pathways [17,18,19,20]; knockout of ADAMTS18 results in spontaneous fibrosis of the submandibular salivary gland in adult mice [21]. Furthermore, the ADAMTSL family, which lacks protease activity, serves as structural components and signaling hubs in the extracellular matrix. For instance, ADAMTSL2 contributes to cardiac repair following myocardial infarction by activating the LRP6/β-catenin signaling pathway [22], and promotes proliferation, migration, and lipid metabolism of colorectal cancer cells via the Notch signaling pathway [23]. In summary, members of the ADAM, ADAMTS, and ADAMTSL families are extensively involved in extracellular matrix remodeling, regulation of signaling pathways, and various pathophysiological processes in either protease-dependent or protease-independent manners.

Although the functions of some members of the above-mentioned family have been elucidated with considerable clarity, the biological functions and mechanisms of action of many others remain to be further explored. ADAMTS16, as a member of the ADAMTS family that has not been thoroughly studied, was first identified by Cal et al. in 2002 using bioinformatics methods [24]. Over the past two decades, only about 70 related articles have been published, and no systematic review has yet summarized its physiological and pathological roles. Current evidence suggests that ADAMTS16 may play important roles in reproductive system development, renal morphogenesis, and cardiovascular diseases. However, its specific substrate repertoire, regulatory networks, and pathophysiological significance still need to be elucidated. Therefore, on the basis of summarizing the ADAMTS family, this review mainly focuses on the potential roles of ADAMTS16 in various tissues and related diseases, aiming to provide a reference for future research.

## 2. The ADAMTS Family

**Figure 2:**
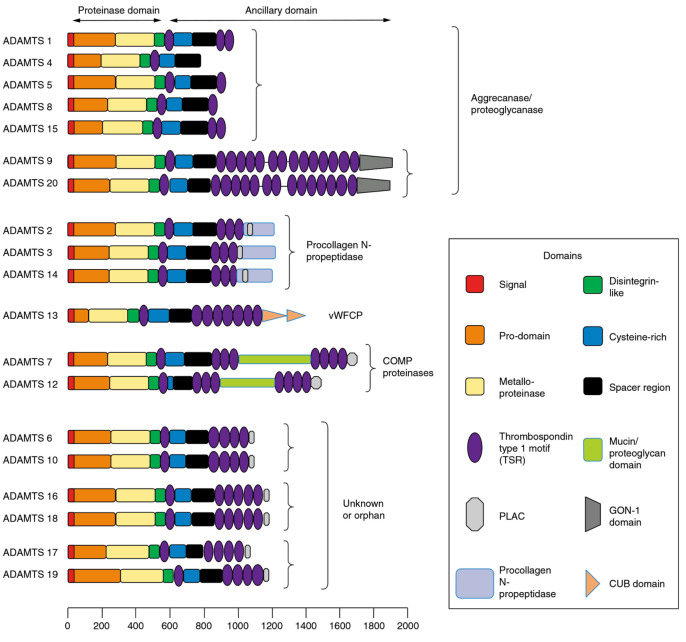
Schematic diagram of the ADAMTS family structure. The abbreviation vWFCP refers to von Willebrand factor-cleaving protease (ADAMTS13). In the figure, signal denotes the signal peptide.

Based on substrate specificity and enzymatic activity, the 19 members of the ADAMTS family are traditionally divided into five classes [25]: the proteoglycanases (ADAMTS1, 4, 5, 8, 9, 15, 20), the procollagen N-peptidases (ADAMTS2, 3, 14), the von Willebrand factor-cleaving protease (ADAMTS13), the cartilage oligomeric matrix protein-cleaving enzymes (ADAMTS7, 12), and the orphan enzymes (ADAMTS6, 10, 16, 17, 18, 19) (Figure 2). ADAMTS4 and ADAMTS5 are relatively well-studied members of the proteoglycanases. By degrading aggrecan in articular cartilage, they serve as key enzymes responsible for cartilage destruction in osteoarthritis (OA). Consequently, the development of inhibitors targeting ADAMTS4/5 has become a hot topic in drug development for osteoarthritis [26,27,28]. Furthermore, ADAMTS5 is widely expressed in various tissues and is also involved in multiple pathological processes, including vascular calcification, cancer cell invasion, and inflammation [29,30,31,32]. ADAMTS2 is a representative member of the procollagen N-peptidases. It participates in the maturation of fibrillar collagens and is associated with processes such as angiogenesis, lymphangiogenesis, neural development, immunity, and spermatogenesis [33]. Emerging evidence indicates that silencing ADAMTS2 alleviates fibroblast-mediated fibrosis through paracrine signaling and promotes axonal regeneration in vitro [34]. Von Willebrand factor (vWF) is a physiological substrate of ADAMTS13. ADAMTS13 regulates the coagulation process by cleaving vWF, and its functional deficiency leads to abnormal accumulation of vWF multimers, thereby inducing thrombotic thrombocytopenic purpura (TTP) [35,36]. Consequently, extensive research has focused on ADAMTS13 antibodies, aiming to improve TTP and other thrombotic indications [37]. In addition, recent findings reveal that ADAMTS13 may circulate in a truncated, hyperactivated form in some trauma patients, thereby playing a novel role in severe traumatic injury [38]. ADAMTS7 is a representative member of the cartilage oligomeric matrix protein (COMP)-cleaving enzymes. Under physiological conditions, ADAMTS7 directly binds to COMP and participates in the dynamic remodeling of the cartilage matrix via COMP cleavage. Under pathological conditions such as OA and rheumatoid arthritis (RA), its abnormally elevated expression leads to excessive degradation of COMP, thereby disrupting the structural integrity of the cartilage extracellular matrix [39,40]. In recent years, however, research on ADAMTS7 has primarily focused on its role in the cardiovascular system. Overexpression of *ADAMTS7* promotes atherosclerotic burden and aortic foam cell formation, and its expression is not restricted to a single vascular cell type [41]. Moreover, *ADAMTS7* and *ADAMTS12* are co-expressed during heart valve development and function cooperatively to prevent myxomatous valve degeneration [42].

Among the ADAMTS family, the “orphan enzymes”—ADAMTS6 and ADAMTS10 are sister proteases, exhibiting high amino acid sequence similarity (approximately 60% identity) in both their full-length and catalytic domains. ADAMTS17 and ADAMTS19 are more closely related to each other (56% identity) [43]. ADAMTS16 and ADAMTS18 share 57% overall amino acid sequence identity, with their catalytic domains reaching up to 85% similarity [24]. Unlike other family members, the physiological substrates of these “orphan enzymes” remained elusive for a long time, and their functions were obscure. However, with advancing research, their substrates have gradually come to light. ADAMTS6 can cleave LTBP1, Syndecan 4, and certain microfibrils [44]. In addition, ADAMTS6 has also been shown to bind and cleave fibrillin-1, fibrillin-2 and fibronectin [45]. Upon activation by furin, ADAMTS10 can cleave fibrillin-2 [46]. Furthermore, mass spectrometry analysis of cell-free extracellular matrix (ECM) in vitro identified fibronectin (FN) as a novel substrate of ADAMTS16 [47]. ADAMTS17 is closely associated with FBN1, yet its substrate and biological function remain unclear [48]. ADAMTS18 specifically cleaves fibronectin (FN) at the A292–V293 site, generating a 29 kDa fragment [49], whereas the substrate of ADAMTS19 remains unknown. Overall, ADAMTS6, 10, and 17 are primarily associated with fibrillin, whereas ADAMTS16 and 18 are linked to fibronectin. Consequently, these “orphan enzymes” can be regarded as ADAMTS proteases related to fibrillin and fibronectin [50]. Such similarity in amino acid sequences and substrates may imply functional relationships between each pair of homologous sister proteases, wherein “homologous sisters” often exhibit redundant or overlapping functions. Taking ADAMTS16 and ADAMTS18 as examples, the two proteases exhibit highly similar functions, both participating in the maintenance of matrix homeostasis and the morphogenesis and development of reproductive system organs. ADAMTS18 is highly expressed in the developing prostate, basal cells of the preputial gland, and vaginal epithelium; its deficiency impairs normal morphogenesis of the preputial gland in male mice [51]. Similarly, ADAMTS16 also plays an important role in reproductive development [52]. Furthermore, both proteases have been validated as pro-fibrotic factors in pathological fibrosis models of the heart and kidney via latent TGF-β activation [53,54,55]. Despite their functional convergence in activating latent TGF-β, ADAMTS16 and its sister protease ADAMTS18 exhibit distinct regulatory mechanisms and tissue specificity. ADAMTS18, but not ADAMTS16, is directly bound and regulated by TCF4 at its promoter. Furthermore, *Adamts18* deficiency in the unilateral ureteral obstruction (UUO) model does not result in compensatory upregulation of *Adamts16*, suggesting that these two proteases operate independently without transcriptional adaptation [55]. Beyond fibrosis, *ADAMTS16* and *ADAMTS18* display complementary rather than redundant distribution patterns in developing organs. *ADAMTS16* is more preferentially involved in urogenital development (including kidney branching morphogenesis, testicular development, and follicular maturation) and male fertility, whereas *ADAMTS18* is more specialized in ocular development, renal immune microenvironment regulation, lung branching morphogenesis, and mammary stem cell niche maintenance [56,57,58]. Moreover, along with *ADAMTS1*, *ADAMTS7*, and *ADAMTS18*, *ADAMTS16* participates in the regulation of TGF-β signaling by directly binding to latency-associated peptide (LAP) and inducing conformational changes that release active TGF-β [53,54,55,59,60]. Collectively, understanding the function of ADAMTS16 requires placing it within the broader context of the ADAMTS protease family, where ADAMTS6, 10, 16, 17, 18, and 19 are intimately interconnected and play critical roles in ECM remodeling, organ morphogenesis, and TGF-β signaling regulation.

## 3. ADAMTS16 Gene, Protein Structure, and Expression

**Figure 3:**
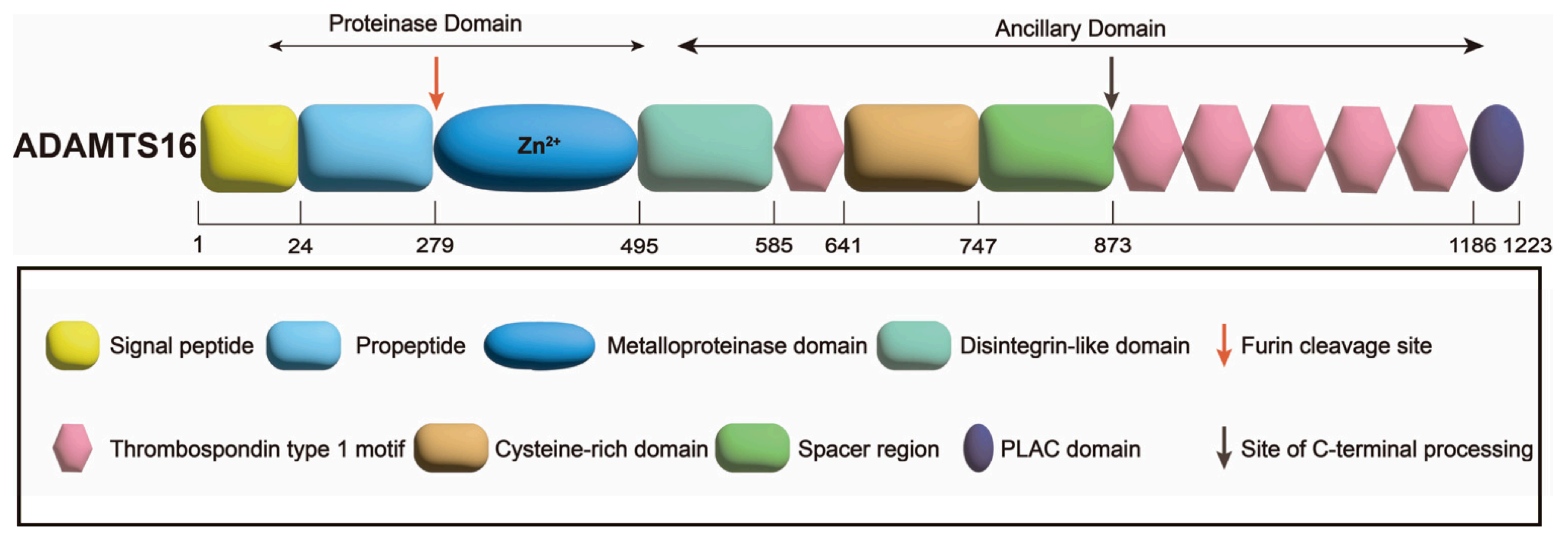
Schematic diagram of the ADAMTS16 structure.

*ADAMTS16* is located on the long arm of human chromosome 5 (5p15), a region devoid of clustering with other ADAMTS family members, and has a full length of 3675 bp [24]. As a secreted metalloproteinase, its domain architecture exhibits typical features, consisting sequentially from the N-terminus to the C-terminus of a signal peptide, a propeptide, a metalloproteinase catalytic domain, a disintegrin-like domain, a central thrombospondin type I motif (TSR I), a cysteine-rich domain, a spacer domain, five C-terminal thrombospondin type I repeats (TSR I repeats), and a PLAC domain (Figure 3).

**Figure 4:**
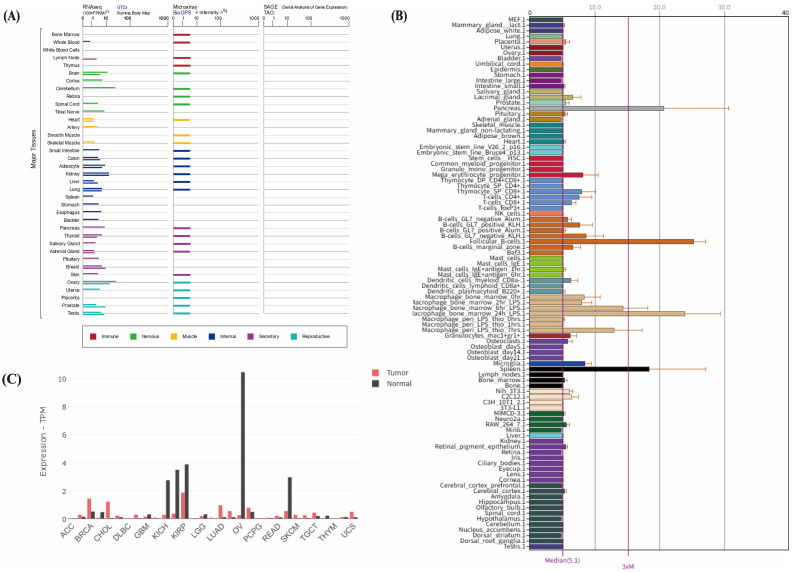
Expression profiles of ADAMTS16 across different species. (**A**) ADAMTS16 expression in human normal tissues, derived from the GeneCards database; (**B**) ADAMTS16 expression in mouse tissues, derived from the BioGPS database; (**C**) ADAMTS16 expression in tumor tissues, derived from the GEPIA2 database. The horizontal axis denotes standard TCGA cancer abbreviations. Key abbreviations: breast invasive carcinoma (BRCA), cholangiocarcinoma (CHOL), kidney chromophobe (KICH), kidney renal papillary cell carcinoma (KIRP), lung adenocarcinoma (LUAD), ovarian serous cystadenocarcinoma (OV), pheochromocytoma and paraganglioma (PCPG), rectum adenocarcinoma (READ).

Under normal physiological conditions, *ADAMTS16* mRNA is expressed in human fetal lung, kidney, and liver tissues, as well as in adult brain, prostate, and ovary [24,61]. In the ovary, *ADAMTS16* is mainly expressed in the parietal granulosa cells of preovulatory follicles. In normal colorectal tissues, *ADAMTS16* is highly expressed, with particularly high expression levels observed in the goblet cells of the crypts and in colonic epithelial cells [62]. Additionally, transcripts of *ADAMTS16* have been identified in human cartilage, pancreas, and spinal cord [61,63,64]; however, the precise cellular localization of this gene across different tissues remains to be fully elucidated. Overall, *ADAMTS16* is mainly expressed in the human reproductive system (ovary), nervous system (brain, cerebral cortex, cerebellum, retina, spinal cord), and multiple visceral organs (kidney, lung, adipose tissue, colon) (Figure 4A). Similar to human *ADAMTS16*, rat *ADAMTS16* is expressed in tissues including the kidney, thymus, lung, heart, liver, and spleen, with particularly prominent expression in the kidney [65]. In Yanshan cashmere goats, the expression level of ADAMTS16 was significantly higher in the ovaries and uterus than in other organs [66]. In mice, the ADAMTS16 protein was detected in the testes and ovaries, with predominant localization in granulosa cells and ovarian surface mesothelial cells [67,68]. An analysis of G protein-coupled receptor expression profiles in mouse macrophages revealed that *ADAMTS16* exhibited extremely significantly high expression in follicular B-cells, microglia, and the prostate. Meanwhile, *ADAMTS16* also showed significantly high expression in immune cell populations, as well as in cells and tissues including the lacrimal gland, placenta, and retinal pigment epithelium [69] (Figure 4B).

Under pathological conditions, the expression of *ADAMTS16* is elevated in osteoarthritic (OA) cartilage and synovial tissues compared to normal tissues [70]. It is upregulated in esophageal squamous cell carcinoma (ESCC), thoracic aortic aneurysm and dissection tissues, non-small-cell lung cancer (NSCLC), renal cell carcinoma (RCC), and gastric cancer, whereas it is downregulated in colorectal cancer and insulin-dependent chondrosarcoma [62,64,71,72,73,74]. In breast invasive carcinoma (BRCA), cholangiocarcinoma (CHOL), and lung adenocarcinoma (LUAD), *ADAMTS16* expression levels are also significantly higher than those in corresponding normal tissues. Notably, consistent with the aforementioned findings, *ADAMTS16* is abundantly expressed in normal genitourinary tissues such as the ovary and kidney but is significantly downregulated in the corresponding tumors, suggesting that its function is highly focused on the genitourinary system (Figure 4C).

## 4. ADAMTS16 Isoforms and Truncated Fragments

An isoform is defined as a transcript exhibiting a unique sequence that originates from the same gene through mechanisms such as alternative splicing or post-translational modification. Currently, the NCBI Gene database shows that *ADAMTS16* has a total of five isoforms, with accession numbers: NM_139056.4, XM_047416874.1, XM_054351924.1, XM_047416875.1, and XM_054351925.1. Furthermore, an additional expressed sequence tag (EST), designated AK122980, was identified in a study that utilized the *ADAMTS16* cDNA sequence as a probe to search the EST database; this sequence precisely aligns with nucleotides 941 to 1229 of *ADAMTS16* [61]. In vitro immunoblotting experiments have revealed multiple bands of ADAMTS16 protein, with apparent molecular sizes of 137 kDa, 65 kDa, 43 kDa, and 40 kDa [71,73,74]. The 137 kDa band corresponds to the full-length ADAMTS16 protein; therefore, the 65 kDa, 43 kDa, and 40 kDa bands are speculated to represent different isoforms. ADAMTS proteases are initially synthesized as inactive zymogen precursors. At the conserved furin cleavage sequence R-X-R/K-R (Arg-Xaa-Arg/Lys-Arg) site in humans, furin convertase proteolytically removes the propeptide, exposing the catalytic domain and thereby converting the enzyme into its active, mature protease form. As a member of the ADAMTS family, ADAMTS16 similarly relies on furin and furin-like proprotein convertases to cleave the propeptide at the N-terminus, thereby mediating the secretion of the mature protein [24].

Studies have found that rat ADAMTS16 differs from human ADAMTS16 by three amino acids in length, with a key variation located at the putative furin cleavage site. To test whether this site variation affects furin cleavage efficiency, the investigators constructed a recombinant protein, Adamts16Pro-Cat, comprising the propeptide and catalytic domains, and showed that this variation did not significantly affect furin cleavage efficiency [65]. To explore the potential function of an alternative form of ADAMTS16, the trADAMTS-16F647 recombinant protein was synthesized and assessed its glycosaminoglycan (GAG) binding affinity and enzymatic activity; the results showed that trADAMTS-16F647 possessed neither effective GAG binding affinity nor aggrecanase activity [75]. Furthermore, the His357 residue in the metalloprotease domain of ADAMTS16 is highly conserved across multiple species and is located adjacent to the functionally critical S2’ loop. To validate the functional importance of this residue, researchers generated a point mutant ADAMTS16-SH (H357Q) and a proteolytically inactive mutant ADAMTS16-SH (E434A), and incubated them with 70 kDa fibronectin to assess enzymatic activity. The results showed that the fibronectin cleavage fragment (30 kDa) was barely detectable in the ADAMTS16-SH (H357Q) mutant, and was completely absent in the ADAMTS16-SH (E434A) mutant, indicating that His357 is a critical residue for maintaining the catalytic function of ADAMTS16 [76]. Furthermore, the hypervariable loops within the spacer domain of the ADAMTS family may also play an important regulatory role in its hydrolytic activity [77].

## 5. The Role of ADAMTS16 in Physiology and Pathology

**Figure 5:**
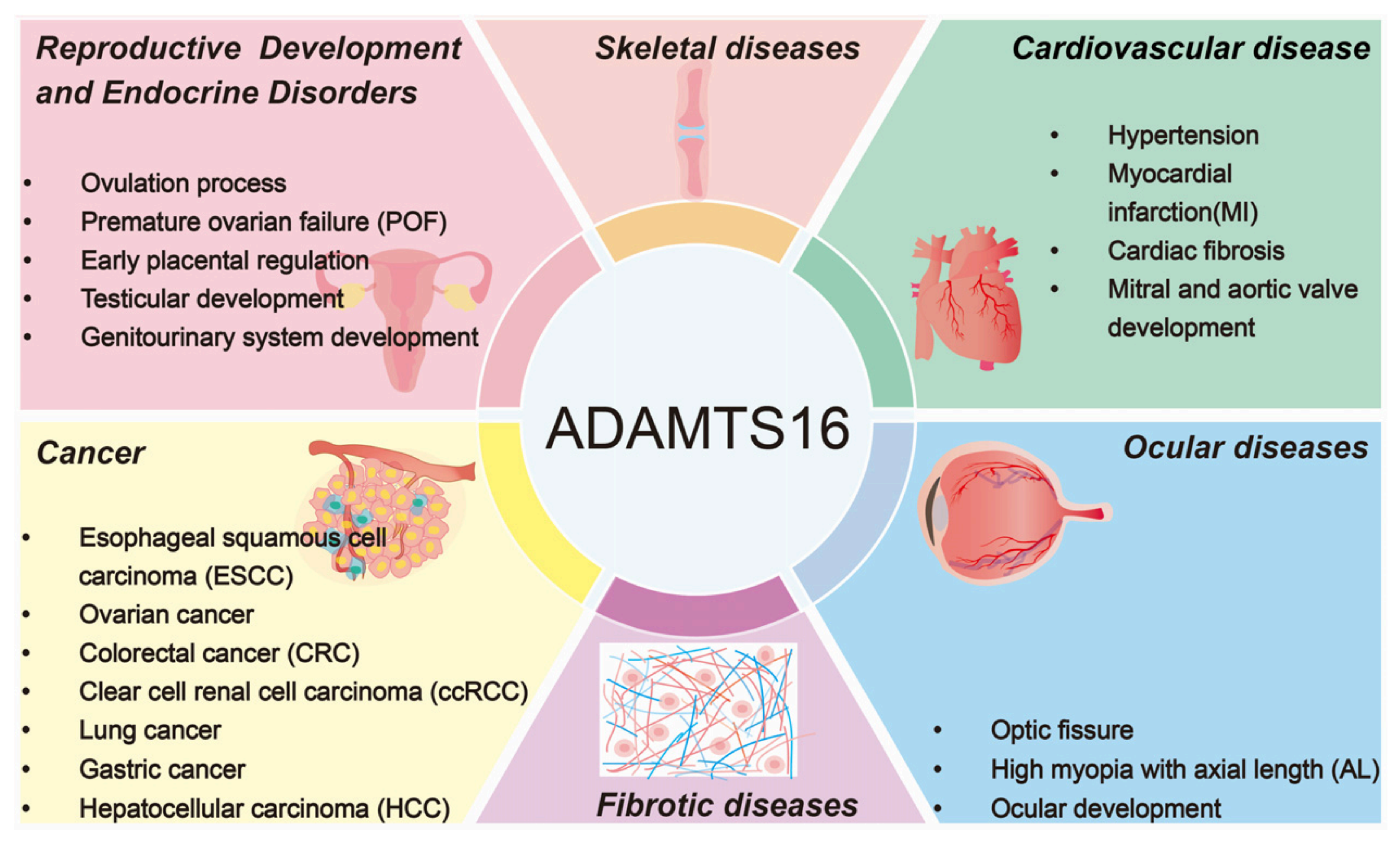
Overview of research on ADAMTS16-related diseases.

ADAMTS16 exhibits multi-tissue distribution and performs tissue-specific functions, primarily involving the reproductive system, cardiovascular diseases, ocular development and pathologies, skeletal disorders, cancer, and fibrotic diseases (Figure 5).

### 5.1. Reproductive Development and Reproductive Endocrine Disorders

In recent years, multiple lines of evidence have indicated that ADAMTS16 plays important physiological roles in the development of the reproductive organ system. In the ovary, ADAMTS16 is primarily expressed in the mural granulosa cells of preovulatory follicles, whereas its expression levels are relatively low in cumulus cells. In fully differentiated luteinized granulosa cells, FSH and forskolin can induce the expression of *ADAMTS16*, suggesting that its transcription is regulated by the cAMP signaling pathway. Furthermore, ADAMTS16 has been demonstrated to cleave α2-macroglobulin (MG), a substrate abundantly present in ovarian follicular fluid [61]. These findings confirm that ADAMTS16 is an active protease and exerts physiological roles in ovarian follicles, at least during the preovulatory stage [61]. Subsequent studies have further demonstrated that the synergistic interaction between thyroid-stimulating hormone, thyroglobulin, and the *ADAMTS16* gene suggests its potential involvement in the pathogenesis of premature ovarian insufficiency (POI) by regulating ovarian estradiol levels [78,79]. By comparing the genome-wide DNA methylation profiles of placental tissues from four groups—in vitro fertilization (IVF)-term delivery, IVF-preterm delivery, natural conception-term delivery, and natural conception-preterm delivery—it was found that *ADAMTS16* expression levels showed no significant difference between term and preterm placentas. However, its expression was significantly elevated during the first and second trimesters of pregnancy, with predominant localization in the embryo and syncytiotrophoblast layers. Further in vitro functional assays confirmed that knockdown of *ADAMTS16* led to reduced invasiveness of extravillous trophoblast (EVT) cells and decreased matrix metalloproteinase (MMP) activity, indicating that ADAMTS16 plays an important role in the regulation of early placental development and susceptibility to preterm birth (PTB) [80].

Furthermore, a deficiency of *ADAMTS16* leads to cryptorchidism and infertility in rats. Masson’s trichrome staining revealed abnormal accumulation of collagen and extracellular matrix (ECM) in the periumbilical region of *ADAMTS16*-deficient rats, suggesting that ADAMTS16 may facilitate the extension of the gubernaculum from the umbilicus toward the scrotum by degrading ECM in the inguinal fat pad (IFP) [52]. In contrast, ADAMTS16-deficient male mice, although retaining fertility, exhibit reduced testicular weight, indicating that this gene is not essential for testicular development in mice but may play a role in gonadal sex determination [68]. Another study has reported that Adamts16 and Wilms’ tumor suppressor protein (Wt1) are both expressed in XY and XX embryonic gonads, with more pronounced expression in the embryonic testis, and they display a co-expression pattern. Further investigations demonstrated that *Adamts16* expression is regulated by Wt1, suggesting that Adamts16 may be involved in the development of the murine genitourinary system [67]. It should be noted that most of the above studies were conducted using rat or mouse models; whether ADAMTS16 exerts similar functions in humans remains unclear and warrants further investigation.

### 5.2. Cardiovascular Diseases

Cardiac development encompasses processes such as migration of myocardial precursor cells, neural crest cell migration, cardiac looping, and formation of cardiac valves and chambers, accompanied by dynamic remodeling of the extracellular matrix (ECM). Following cardiac injury, ECM deposition and subsequent scar formation represent key events in both physiological repair and pathological processes [81]. ADAMTS16 participates in ECM degradation and remodeling, thereby playing important roles in cardiovascular diseases, including hypertension, myocardial infarction, cardiac fibrosis, and bicuspid aortic valve, by regulating cellular behavior. Early studies have reported that ADAMTS16 is associated with blood pressure regulation, contributing to blood pressure control through its effects on aortic pulse wave velocity, vascular medial thickness, glomerular filtration rate, and renal hemodynamics [65,82]. Western blot analysis revealed that ADAMTS16 expression was upregulated in tissues of thoracic aortic aneurysm and dissection [71]. Studies have shown that in pressure overload-induced cardiac hypertrophy and heart failure (HF) models, ADAMTS16 expression is elevated and promotes cardiac fibroblast activation through interaction with the LAP-TGF-β signaling pathway, thereby exacerbating cardiac fibrosis, myocardial hypertrophy, and the progression of heart failure [53]. In a rat model of myocardial infarction, miR-29b-3p expression was downregulated, whereas exogenous supplementation with miR-29b-3p improved hemodynamics, reduced collagen volume fraction, increased vascular density, and attenuated cardiomyocyte injury [83]. Conversely, upregulation of *ADAMTS16* expression attenuates the cardioprotective effect of miR-29b-3p [83]. Additionally, in situ hybridization and lineage tracing studies have revealed that ADAMTS16 exhibits a unique spatiotemporal expression pattern during aortic valve development. Deficiency of *ADAMTS16* leads to increased expression of extracellular matrix components (including fibronectin, aggrecan, and collagen IV) in bicuspid aortic valves and induces valve thickening, aortic valve stenosis, and other BAV-related phenotypes [76].

### 5.3. Eye-Related Diseases

During vertebrate eye development, the closure of the optic fissure (OF) is crucial for the formation of the structural integrity of the eye [84]. RNA in situ hybridization (ISH) results revealed that *ADAMTS16* mRNA is expressed at the margins of the closing optic fissure in both mice and zebrafish. Knockdown of *ADAMTS16* led to persistent openness of the optic fissure in zebrafish, whereas rescue experiments successfully restored the coloboma phenotype [85]. It has been reported that, compared with wild-type human-induced pluripotent stem cells, the secretome of cells deficient in the C-mannosyltransferases DPY19L1 or DPY19L3 lacks multiple ADAMTS metalloproteinases, including ADAMTS16 [86]. Following in vivo targeted knockout of DPY19L1 or DPY19L3, zebrafish exhibited impaired optic fissure closure, a phenotype similar to that induced by ADAMTS16 knockdown, leading to the hypothesis that ADAMTS16 is a target of C-mannosylation. THBS1 is a known and validated C-mannosylation target protein whose TSRs contain three conserved tryptophan motifs (WXXWXXWXXWXXC) that serve as C-mannosylation sites [87,88]. Mass spectrometry analysis further confirmed that the WSDWSSWSPC sequence within the ADAMTS16 TSR1 domain serves as a C-mannosylation site. Of note, a genome-wide association study (GWAS) identified the *ADAMTS16* gene locus (rs7704912) as a novel candidate locus for axial length (AL) in high myopia. RT-qPCR and Western blot analyses revealed that the expression level of ADAMTS16 was significantly upregulated in the neural retina of an experimental myopia mouse model [89].

### 5.4. Skeletal Diseases

Cartilage tissue predominantly comprises chondrocytes and the extracellular matrix (ECM), which function synergistically to preserve cartilage homeostasis. The ECM of cartilage is composed of collagen, proteoglycans, and non-collagenous proteins such as chondronectin and fibronectin, as well as components including cartilage oligomeric matrix protein (COMP) [90,91]. Chondrogenesis refers to the process whereby mesenchymal cells aggregate, differentiate into chondrocytes, and secrete substantial quantities of ECM, culminating in the formation of cartilage tissue.

Empirical evidence indicates that the expression levels of ADAMTS16, and furin are upregulated during this process [92]. Further investigations have revealed that ADAMTS16 expression is also upregulated in osteoarthritic (OA) cartilage and synovial tissues [63,79,93]. Notably, the expression level of matrix metalloproteinase 13 (MMP13) was also elevated in the aforementioned tissues and was considerably higher than that of ADAMTS16 [70]; however, upon *ADAMTS16* overexpression, *MMP13* expression was conversely reduced [93]. This apparent paradox suggests that other regulatory factors may exist within the osteoarthritic cartilage microenvironment that may mitigate the inhibitory effect of ADAMTS16 on MMP13 production, warranting further investigation. In addition to osteoarthritis, ADAMTS16 has also been associated with other skeletal-related phenotypes. A meta-analysis of genome-wide association studies (GWASs) revealed that *ADAMTS16* and *RUNX1* are associated with the femoral neck length phenotype of the hip joint [94]. Through transcriptomic bioinformatics analysis of expression profiles from the Gene Expression Omnibus (GEO) database, *ADAMTS16* was identified as a potential gene associated with heterotopic ossification (HO) [95]. Collectively, these findings suggest that ADAMTS16 may play important roles in bone development and related pathological processes.

### 5.5. The Relationship Between ADAMTS16 and Cancer

The occurrence and progression of cancer are characterized by malignant biological behaviors, including proliferation, migration, and invasion of cancer cells. Loss of *ADAMTS16* function due to deficient expression, mutations, or promoter hypermethylation can promote tumor formation and disease development. In esophageal squamous cell carcinoma (ESCC), *ADAMTS16* expression is upregulated and has been identified as a key prognostic gene [96]; knockdown of ADAMTS16 inhibits cell growth and invasive capacity [63]. Similarly, *ADAMTS16* missense mutations suppress the growth, migration, and invasion of ovarian cancer cells and increase their sensitivity to cisplatin [97]. In colorectal cancer (CRC), immunohistochemical results have shown down-regulation of *ADAMTS16* expression, and ectopic overexpression of *ADAMTS16* inhibits the growth of colorectal cancer cells [62]. ADAMTS16 exerts a similar role in clear cell renal cell carcinoma (ccRCC), suggesting its potential as a prognostic biomarker for ccRCC [98]. Subsequent studies have confirmed that *ADAMTS16* may be a target gene of miR-25, which negatively regulates *ADAMTS16* expression via the Wnt/β-catenin signaling pathway and participates in the progression of renal cell carcinoma [99].

Epigenetic modifications, notably the hypermethylation of CpG island promoters, represent frequent occurrences in oncogenesis [100]. Studies have demonstrated that the *ADAMTS16* promoter exhibits hypermethylation in epithelial-derived malignancies such as colorectal cancer (CRC), lung cancer (LC), and oral squamous cell carcinoma (SCC) [62,98], and its methylation level is significantly associated with lung cancer risk [101]. Multiple studies have shown that *ADAMTS16* expression is upregulated in lung cancer [72,102,103], where it forms a positive feedback loop with the TGF-β1/SOX4 axis to promote epithelial–mesenchymal transition (EMT) and cell migration in lung adenocarcinoma (LUAD), suggesting its potential as a therapeutic target [72]. Additionally, ADAMTS16 modulates IFI27 through the NF-κB pathway, promoting migration, invasion, and proliferation of gastric cancer cells [74]. In tuberculosis-associated hepatocellular carcinoma (HCC), overexpression of *ADAMTS16* promotes liver cancer progression [104]. Integrated whole-exome and whole-transcriptome sequencing analyses revealed that *ADAMTS16* is associated with the entire dynamic evolution of lung adenocarcinoma (LUAD) from adenocarcinoma in situ (AIS) to microinvasive adenocarcinoma (MIA) [105]. In summary, these findings indicate that ADAMTS16 exhibits functional heterogeneity across different cancer types, exerting both oncogenic and tumor-suppressive effects.

### 5.6. ADAMTS16 in Other Diseases

In addition to the aforementioned diseases, ADAMTS16 has also been associated with bipolar disorder, type 2 diabetes mellitus (T2DM), and fibrotic diseases such as Dupuytren’s contracture and renal fibrosis [54,106,107,108,109]. A genome-wide association analysis of four T2DM-related traits (fasting glucose, fasting insulin, insulin resistance, and β-cell function) revealed that the ADAMTS16 gene locus (rs74806991) is significantly associated with HOMA-IR [108]. Regarding renal fibrosis, ADAMTS16 plays a critical pro-fibrotic role by activating the TGF-β/Smad signaling pathway. Specifically, ADAMTS16-activated TGF-β induces the release of inflammatory cytokines in the renal interstitium, promoting the infiltration of macrophages and neutrophils, while these infiltrating inflammatory cells further release pro-fibrotic factors, thereby establishing a vicious “inflammation–fibrosis” cycle that accelerates renal tissue injury [54]. Furthermore, knockdown of ADAMTS16 leads to elevated expression levels of fibrin and type I collagen [54].

## 6. Regulatory Network and Function of ADAMTS16

**Figure 6:**
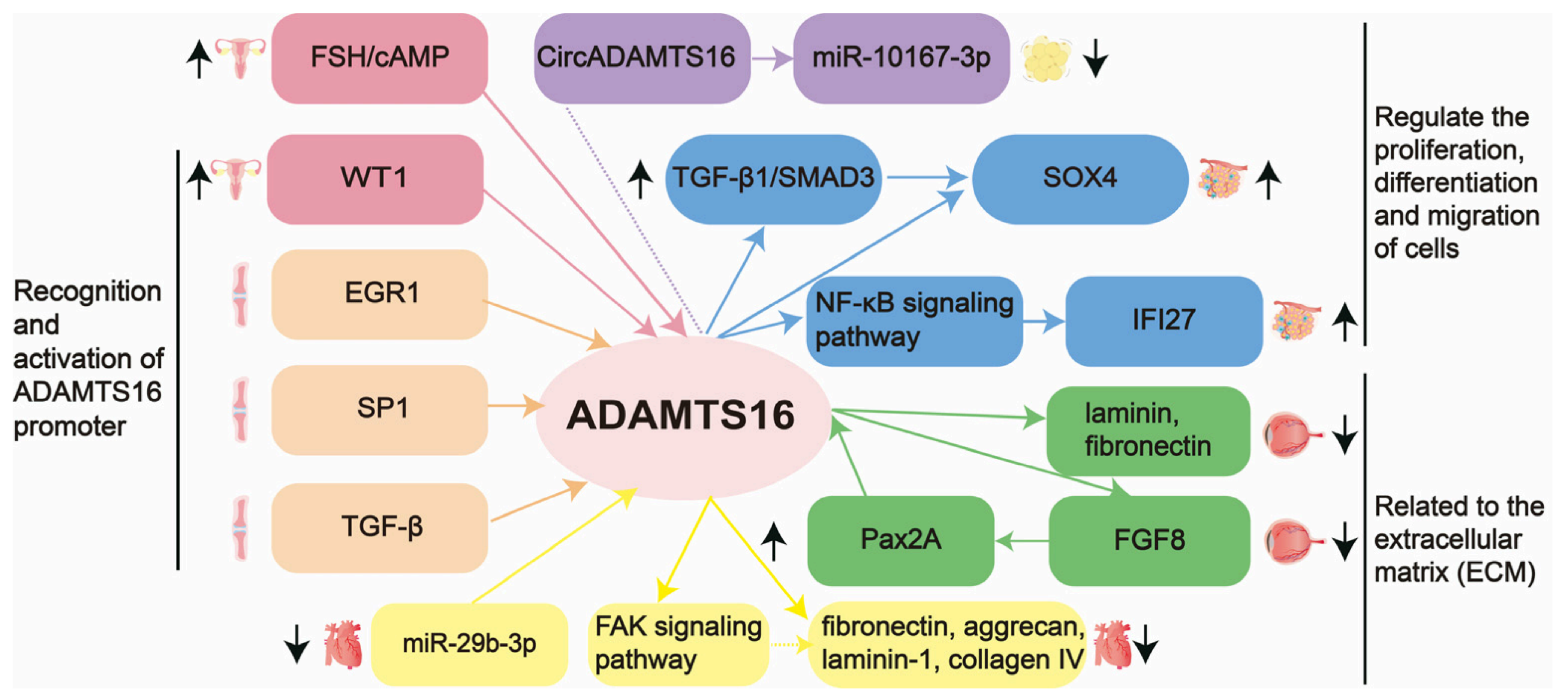
Schematic diagram of the ADAMTS16 regulatory network. Abbreviations: follicle-stimulating hormone (FSH), Wilms’ tumor suppressor protein (WT1), early growth response 1 (EGR-1), Specificity Protein 1 (SP1), transforming growth factor beta (TGF-β), SRY-Box Transcription Factor 4 (SOX4), interferon alpha inducible protein 27 (IFI27), paired box 2A (Pax2A), fibroblast growth factor 8 (FGF8).

The ADAMTS16 protease performs essential functions in development and homeostasis, and its biological activities depend on a sophisticated regulatory network involving multiple proteins or transcription factors (Figure 6). Previous studies have confirmed that ADAMTS16 expression is induced by the FSH/cAMP pathway in granulosa cells [61]; it is regulated by WT1 during embryonic kidney development [67]; and the transcription factors EGR1, SP1, and TGF-β can recognize and activate the *ADAMTS16* promoter in chondrocyte cell lines, positively regulating its expression [93]. Further studies have revealed that ADAMTS16 participates in multiple positive or negative feedback regulatory loops. In tumor-related models, TGF-β1 regulates ADAMTS16 expression via SOX4, and together they form a positive feedback loop with the TGF-β1/SOX4 axis that drives epithelial–mesenchymal transition (EMT) and cell metastasis [72]. ADAMTS16 also regulates IFI27 through the NF-κB pathway, thereby influencing gastric cancer progression [74]. During eye development, Pax2 drives ADAMTS16 expression, and ADAMTS16 exerts its protease activity to degrade the basement membrane, regulate cell proliferation and apoptosis, and inhibit ectopic Fgf8 expression, thereby controlling the proper closure of the optic fissure (OF). Concurrently, the suppression of Fgf8 by ADAMTS16 maintains normal Pax2 expression levels, establishing a dynamic balance through a negative feedback loop [85]. Additionally, deficiency of *ADAMTS16* results in elevated levels of phosphorylated FAK (p-FAK), fibronectin, aggrecan, laminin-1, and type IV collagen, whereas treatment with a 30 kDa recombinant fibronectin fragment attenuates the FAK signaling pathway [76]. At the level of non-coding RNA regulation, upregulated miR-29b-3p delivered by exosomes secreted from bone marrow mesenchymal stem cells (BMSCs) improves myocardial angiogenesis and ventricular remodeling by targeting ADAMTS16 [83]. In addition, the *ADAMTS16*-related circular RNA, circADAMTS16, is highly expressed in bovine adipose tissue and promotes the proliferation while inhibiting the differentiation of bovine adipocytes through targeted regulation of miR-10167-3p [110]. In summary, the biological functions of ADAMTS16 involve complex regulatory networks, and the underlying molecular mechanisms await further elucidation.

## 7. Therapeutic Potential of ADAMTS16

### 7.1. Biomarker Potential

As a secreted protein, the expression level, epigenetic changes, and genetic variations in *ADAMTS16* are closely associated with disease onset, progression, and prognosis, endowing it with significant potential as a clinical biomarker. In lung adenocarcinoma (LUAD) and gastric cancer (GC), elevated ADAMTS16 expression in tumor tissues correlates with advanced disease stage, lymph node/distant metastasis, and shorter overall survival (OS) and disease-free survival (DFS) [72,74]. Conversely, in colorectal cancer (CRC) and clear cell renal cell carcinoma (ccRCC), low ADAMTS16 expression is associated with higher recurrence rates and poor prognosis [98]. Thus, detection of ADAMTS16 in tissue samples could be used for patient prognostic stratification, highlighting its value as a prognostic biomarker.

Moreover, ADAMTS16 promoter hypermethylation is an early event in multiple epithelial-derived malignancies, including CRC, lung cancer (LC), and oral squamous cell carcinoma (SCC), leading to ADAMTS16 silencing [62,101]. The methylation level of the ADAMTS16 promoter in tissue samples or circulating tumor DNA (ctDNA) holds significant value for early cancer diagnosis and prognosis prediction.

### 7.2. Therapeutic Targeting

To establish ADAMTS16 as a viable therapeutic target, a comprehensive delineation of its substrate repertoire under both physiological and pathological conditions is of paramount importance. However, as a member of the metzincin superfamily, ADAMTS16 exhibits context-dependent proteolytic activity, and its natural extracellular matrix (ECM) as well as non-ECM substrates remain largely unidentified. The functional complexity of ADAMTS16 implies that its inhibition or activation may elicit broad downstream effects, and in the absence of a well-defined substrate network, targeted intervention carries substantial uncertainty and attendant risks. Therefore, prior to the implementation of any rational targeted therapy, elucidation of the ADAMTS16 substrate degradome is an essential prerequisite.

To this end, a multi-pronged bioinformatics-guided approach integrating targeted proteomics can be employed. Techniques such as isobaric tags for relative and absolute quantitation (iTRAQ), tandem mass tag (TMT)-based quantitative labeling, and terminal amine isotopic labeling of substrates (TAILS) for N-terminomics, combined with targeted biochemical and cellular assays, including in vitro protease cleavage experiments, co-expression/knockout cellular systems, and surface plasmon resonance (SPR) analysis, can be used to systematically interrogate ADAMTS16 substrates. Based on this, further targeted strategies are developed from the following aspects:

#### 7.2.1. Direct Targeting of ADAMTS16 Expression and Activity

Inhibiting *ADAMTS16* activity or expression via siRNA/shRNA knockdown or gene mutation shows therapeutic potential in diseases where *ADAMTS16* is overexpressed and acts as an oncogene or pathogenic factor. In in vitro tumor models of esophageal squamous cell carcinoma (ESCC), LUAD, and GC, *ADAMTS16*-targeting siRNA/shRNA effectively downregulates *ADAMTS16* expression, promotes apoptosis, and inhibits cell migration and metastasis [64,72,74]. Additionally, *ADAMTS16* mutations (including *ADAMTS16*) are independent predictors of OS and PFS in high-grade serous ovarian cancer, and *ADAMTS16* missense mutations significantly increase ovarian cancer cell sensitivity to cisplatin, improving treatment outcomes [97].

#### 7.2.2. Targeting Downstream Pathways

Interventions targeting downstream regulatory pathways have shown promise. ADAMTS16 primarily activates latent TGF-β via its RRFR motif, triggering the TGF-β/Smad2/3 signaling cascade and promoting cardiac and renal fibrosis as well as EMT-mediated metastasis in LUAD. Additionally, ADAMTS16 cleaves fibronectin to release a 30 kDa fragment, affecting ECM remodeling. Several therapeutic strategies targeting these pathways have been experimentally validated. For instance, the TGF-βR1 inhibitor SB431542 blocks the ADAMTS16/TGF-β1/SOX4 positive feedback loop and inhibits LUAD metastasis [72]; TGF-β neutralizing antibodies block ADAMTS16-induced cardiac fibrosis [53]. Furthermore, the traditional Chinese medicine formula Zhenwu Decoction (ZWD), which significantly reduced ADAMTS16 mRNA expression in the kidneys of UUO rats, presumably by binding ADAMTS16 and inhibiting its downstream TGF-β/Smad pathway [111]. Notably, given the dual functionality of ADAMTS16 across different diseases, targeting its downstream pathways requires careful consideration of disease specificity.

#### 7.2.3. Epigenetic Drug Regulation

ADAMTS16 exhibits promoter hypermethylation in epithelial-derived malignancies including CRC, LC, and SCC [62,101], and its methylation level is significantly associated with lung cancer risk [102]. The demethylating agent 5-Aza-dC has been shown to restore expression of other ADAMTS family members, such as ADAMTS5, ADAMTS9, ADAMTS18, and ADAMTS19, thereby inhibiting tumor cell migration and invasion while promoting apoptosis, indicating restoration of tumor suppressor functions [112,113,114,115]. By extension from these findings on other ADAMTS family members, it is plausible that 5-Aza-dC may exert similar effects on ADAMTS16, warranting further investigation.

## 8. Conclusions and Discussion

This review delineates the expression patterns of ADAMTS16 across a wide range of tissues and cell types, as well as its roles under various physiological and pathological conditions, indicating that the expression patterns and mechanisms of action of this protease vary significantly across different tissues. Based on current research findings, it is speculated that ADAMTS16 may directly or indirectly interact with extracellular matrix (ECM) components to regulate cell growth, differentiation, and migration; and it may also modulate numerous cellular functions through synergistic interactions with other cytokines. Nonetheless, the precise roles of ADAMTS16 remain incompletely understood, warranting further investigation with a focus on specific areas outlined herein.

Identification of substrates and proteolytic activity. Currently, only limited evidence has demonstrated that ADAMTS16 can cleave the protease inhibitor α2-macroglobulin and fibronectin. Nonetheless, multiple factors collectively contribute to the difficulty in substrate identification. Some substrates are expressed only in specific tissues or developmental stages and are finely regulated by the microenvironment. Certain substrates exhibit expression restricted to particular tissues or developmental stages and are subject to precise regulation by the microenvironment, complicating the replication of in vivo conditions in vitro. Research has demonstrated that the proteolytic sites of certain ADAMTS substrates may only become exposed after the formation of protein complexes. ADAMTS6 can interact with and cleave LTBP1 and LTBP3 [45,116]. Co-expression of LTBP3_CT with the TGFβ1 small latent complex (SLC) accelerated the binding/dissociation rate of ADAMTS6 with LTBP3, suggesting that SLC binding may induce conformational changes in LTBP3, thereby affecting the exposure of ADAMTS6 recognition sites.

Transcriptional adaptation (TA) and regulatory relationships among ADAMTS family members. TA refers to the phenomenon in which, following gene mutation that leads to the degradation of the corresponding mRNA, cells upregulate the transcriptional levels of other “adaptive genes” to compensate for the loss of function, thereby maintaining a wild-type phenotype [117,118]. Adamts12 mRNA is upregulated in *Adamts7*−/− hearts, suggesting its sensitivity to transcriptional adaptation [41]. Knockdown of *ADAMTS10* leads to increased ADAMTS6 expression, whereas *ADAMTS6* knockdown results in decreased ADAMTS10 expression, indicating that these two genes can mutually regulate each other’s expression [45]. ADAMTS6 and ADAMTS10 may exhibit either synergistic or negative regulatory effects depending on the experimental system (in vivo/in vitro) and cell type specificity [119]. ADAMTS16 and ADAMTS18 are sister proteases that possess similar cleavage capabilities toward fibronectin (FN); whether transcriptional adaptation exists between these two proteases remains to be further investigated.

Mechanistic Heterogeneity of ADAMTS16. Despite the growing understanding of ADAMTS16 functions, a fundamental question remains: Why does the same molecule exert opposing roles in different tissues and disease contexts? As summarized throughout this review, ADAMTS16 acts as an oncogene in LUAD, GC, and ESCC, yet functions as a tumor suppressor in CRC and ccRCC. Similarly, it promotes fibrosis in the heart and kidney while playing distinct roles in ovarian and testicular development. Several hypotheses may explain this contextual duality. First, tissue-specific microenvironmental factors, including ECM composition, growth factor availability, and mechanical forces, may dictate ADAMTS16′s substrate preference and downstream signaling outcomes. Second, substrate accessibility may vary across tissues; fibronectin, a validated ADAMTS16 substrate, is differentially expressed and organized in distinct ECM niches. Third, unknown tissue-specific cofactors or post-translational modifications may modulate ADAMTS16’s activity, stability, or interaction partners. These mechanistic uncertainties directly translate into challenges for therapeutic development. To date, there are no reported small-molecule inhibitors specifically targeting ADAMTS16. The RRFR motif, which is critical for latent TGF-β activation, represents a potential target for neutralizing antibodies, but such agents have not yet been developed. Furthermore, tissue-specific drug delivery systems would be essential to avoid off-target effects given ADAMTS16′s dual roles across different organs. Addressing these gaps represents a priority for translating ADAMTS16 biology into clinical applications.

## Data Availability

No new data were created or analyzed in this study. Data sharing is not applicable to this article.
